# Antioxidants in Cardiovascular Health: Implications for Disease Modeling Using Cardiac Organoids

**DOI:** 10.3390/antiox14101202

**Published:** 2025-10-03

**Authors:** Gracious R. Ross, Ivor J. Benjamin

**Affiliations:** Cardiovascular Center, Medical College of Wisconsin, 8701 Watertown Plank Rd., Milwaukee, WI 53226, USA; rossgracious@icloud.com

**Keywords:** cardiac organoids, human iPSCs, disease modeling, redox homeostasis, biomarkers, heart disease, preclinical, drug development, antioxidants, reductive stress

## Abstract

Cardiovascular disease remains the leading cause of mortality worldwide, and at its molecular core lies a silent disruptor: oxidative stress. This imbalance between reactive oxygen species (ROS) and antioxidant defenses not only damages cellular components but also orchestrates a cascade of pathological events across diverse cardiac cell types. In cardiomyocytes, ROS overload impairs contractility and survival, contributing to heart failure and infarction. Cardiac fibroblasts respond by promoting fibrosis through excessive collagen deposition. Macrophages intensify inflammatory responses, such as atherosclerosis, via ROS-mediated lipid oxidation—acting both as mediators of damage and targets for antioxidant intervention. This review examines how oxidative stress affects cardiac cell types and evaluates antioxidant-based therapeutic strategies. Therapeutic approaches include natural antioxidants (e.g., polyphenols and vitamins) and synthetic agents (e.g., enzyme modulators), which show promise in experimental models by improving myocardial remodeling. However, clinical trials reveal inconsistent outcomes, underscoring translational challenges (e.g., clinical biomarkers). Emerging strategies—such as targeted antioxidant delivery, activation of endogenous pathways, and disease modeling using 3D organoids—aim to enhance efficacy. In conclusion, we spotlight innovative technologies—like lab-grown heart tissue models—that help scientists better understand how oxidative stress affects heart health. These tools are bridging the gap between early-stage research and personalized medicine, opening new possibilities for diagnosing and treating heart disease more effectively.

## 1. Introduction

**Figure 1:**
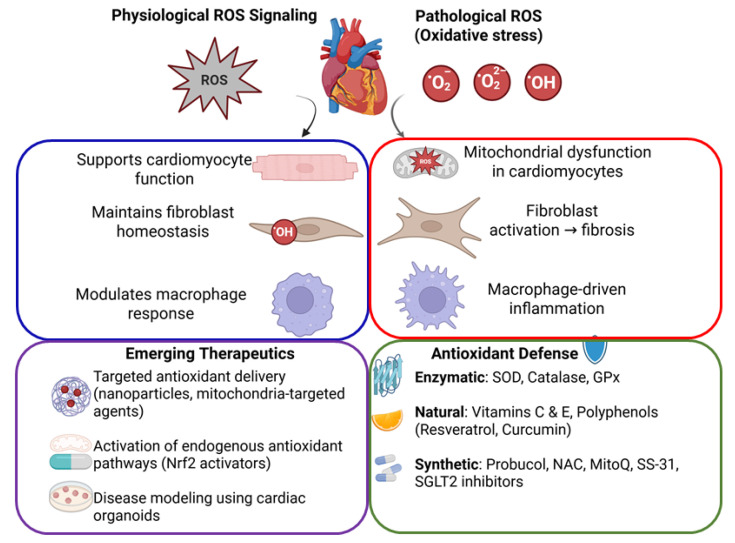
Schematic diagram illustrating oxidative stress and antioxidant mechanisms in cardiovascular health. Balanced ROS signaling supports normal cardiac function, whereas excessive ROS causes cellular damage, leading to cardiac dysfunction, fibrosis, and inflammation. Antioxidant defenses and emerging targeted therapeutic strategies aim to restore cardiovascular homeostasis.

Oxidative stress in the cardiovascular system is defined by an excess of reactive oxygen and nitrogen species (ROS/RNS) relative to antioxidant defenses [1]. At physiological levels, ROS serve as signaling molecules that regulate cellular processes such as growth, differentiation, and apoptosis. However, when ROS generation outpaces antioxidant capacity, highly reactive radicals can oxidize proteins, lipids, and DNA, leading to cellular dysfunction and death [2]. Cardiomyocytes, fibroblasts, and macrophages are the predominant myocardial cells that are all impacted by oxidative stress, albeit in different ways. Cardiomyocytes are highly aerobic muscle cells with abundant mitochondria; excessive ROS in these cells can damage mitochondrial DNA and enzymes, impair calcium handling, and trigger cell death pathways. Fibroblasts, the main producers of the cardiac extracellular matrix, respond to ROS by activating and differentiating into myofibroblasts, which secrete collagen and contribute to fibrosis [3]. Macrophages, the immune cells resident in the heart and vasculature, produce ROS as part of the inflammatory response. In pathological conditions, macrophage-derived ROS oxidize low-density lipoproteins (LDLs) and amplify inflammation, as seen in atherosclerotic plaques [4]. Antioxidants—including enzymatic defenses and dietary molecules—are essential in counteracting these oxidative processes. A robust antioxidant system maintains redox homeostasis and protects cardiac cells, whereas impairment of this system or excessive ROS production leads to tissue damage and contributes to cardiovascular diseases. This review highlights the role of antioxidants in normal cardiac physiology, examines how oxidative stress affects cardiomyocytes, fibroblasts, and macrophages in disease, and discusses emerging technologies (e.g., organoids) and therapeutic interventions to restore redox balance in the heart (Figure 1).

## 2. Physiology of Antioxidants in Cardiac Cells

### 2.1. Role of Antioxidants in Normal Cardiac Function

Under normal conditions, a balance between ROS generation and elimination is critical for heart function [1]. Low levels of ROS act as second messengers in cardiomyocytes and other cardiac cells, modulating signaling pathways that regulate myocardial contraction, vascular tone, and cell growth [1]. For example, hydrogen peroxide (H_2_O_2_) at nanomolar concentrations influences protein phosphorylation and gene expression that support physiological processes like angiogenesis and adaptive hypertrophy [5].

These redox signals are tightly regulated, confined to specific subcellular locales and quickly neutralized to prevent indiscriminate damage [5]. Antioxidants, both endogenous and dietary, maintain this redox equilibrium. In cardiomyocytes, basal ROS produced by mitochondrial respiration are quenched by antioxidant enzymes, ensuring that ROS act in beneficial signaling roles without causing structural damage. Similarly, cardiac fibroblasts and resident macrophages maintain low ROS levels under resting conditions, allowing ROS to participate in cell-to-cell communication and defense signaling. Thus, antioxidants are integral to normal cardiac physiology by fine-tuning ROS to levels that support cellular homeostasis and signaling.

### 2.2. Antioxidant Defense Mechanisms in Cardiomyocytes, Fibroblasts, and Macrophages

Vertebrate cells are equipped with an array of antioxidant defense systems to remove or neutralize ROS [1]. Key enzymatic antioxidants include superoxide dismutases (SODs), catalase, and glutathione peroxidases (GPx), which work in concert to detoxify reactive species. Superoxide anion (O_2_˙^−^), a primary ROS, is dismutated to H_2_O_2_ by SOD enzymes; H_2_O_2_ is then converted to water by catalase and GPx [1]. There are three isoforms of SOD: cytosolic CuZn-SOD (SOD1), mitochondrial Mn-SOD (SOD2), and extracellular SOD (SOD3). In the heart, SOD2 is particularly crucial due to the high rate of mitochondrial ROS production in cardiomyocytes. Notably, mice lacking SOD2 die early from dilated cardiomyopathy, underscoring the importance of mitochondrial antioxidant defense in cardiomyocytes. Catalase and GPx are also expressed in cardiac cells; catalase is found in cytosol and peroxisomes (with high activity in liver and present in heart and macrophages) and efficiently clears H_2_O_2_ [1]. GPx and related peroxiredoxins scavenge peroxides and lipid hydroperoxides, maintaining membrane integrity. Cardiac fibroblasts possess these antioxidant enzymes to safeguard against oxidative stress. Macrophages, which often generate ROS during “respiratory burst” to kill pathogens, concurrently activate self-protective antioxidant pathways. The Nrf2/Keap1 signaling pathway is a master regulator of cellular antioxidants in all these cell types [5]. Under oxidative stress, Nrf2 translocates into the nucleus to induce genes encoding glutathione synthesis enzymes, GPx, SOD, heme oxygenase-1 (HO-1), and other cytoprotective proteins [5]. This inducible response is vital in macrophages to prevent self-damage during inflammation, and in cardiac cells broadly to adapt to increased oxidative load. In summary, cardiomyocytes, fibroblasts, and cardiac macrophages are all armed with robust antioxidant defenses—both constitutive enzymes and stress-inducible systems—that preserve cellular function by limiting oxidative damage in the heart’s normal state.

## 3. Pathological Implications of Oxidative Stress in Cardiovascular Diseases

### 3.1. Oxidative Stress in Cardiomyocytes: Mitochondrial Dysfunction and Cell Death

Elevated oxidative stress has deleterious effects on cardiomyocytes, the working muscle cells of the heart. During conditions such as ischemia–reperfusion injury or heart failure, ROS production in cardiomyocytes increases dramatically, overwhelming antioxidant defenses [2]. A primary source of this excess ROS is the mitochondria. Under stress, the electron transport chain can leak electrons that form superoxide, and dysfunctional mitochondria become both victims and amplifiers of oxidative damage. Mitochondria are a key interface in ROS-induced cardiac injury: they produce ROS and are themselves impaired by ROS, creating a vicious cycle of energy failure and structural damage [2]. Oxidative stress can induce the opening of the mitochondrial permeability transition pore, loss of mitochondrial membrane potential, and release of pro-apoptotic factors, thereby triggering cardiomyocyte apoptosis or necrosis. Indeed, in ischemic myocardium, excessive ROS generation leads to ATP depletion and activates cell death pathways, contributing to infarct size and myocardial cell loss [2]. If ROS are not adequately neutralized, they also oxidize contractile proteins and ion channels in cardiomyocytes, impairing excitation-contraction coupling and contractility. Over time, chronic oxidative stress in cardiomyocytes promotes pathological remodeling of the heart. For example, persistent ROS elevation has been linked to hypertrophic signaling and dilation in heart failure [6]. Cardiomyocyte injury may trigger either senescence or cell death, which reduces contractile mass and precipitates systolic dysfunction. The cumulative effect is progressive cardiac dysfunction, as seen in diseases like dilated cardiomyopathy, myocardial infarction, and heart failure. Thus, excessive ROS in cardiomyocytes leads to mitochondrial dysfunction and cell death, forming a molecular basis for cardiac injury and adverse remodeling in oxidative stress-related heart diseases.

### 3.2. Fibroblast Activation and Oxidative Stress–Induced Cardiac Fibrosis

Cardiac fibroblasts are the principal cells responsible for maintaining the extracellular matrix in the heart, and they become highly active during stress or injury. Oxidative stress is a potent stimulus for fibroblast activation and pathological fibrosis [3]. When exposed to ROS (for instance, during angiotensin II stimulation or post-infarction inflammation), quiescent cardiac fibroblasts can proliferate and differentiate into myofibroblasts. These myofibroblasts secrete excessive collagen and other matrix components, leading to myocardial fibrosis and stiffening of cardiac tissue. Elevated ROS levels trigger profibrotic signaling cascades; for example, oxidative stress can activate latent transforming growth factor-β (TGF-β), a master regulator of fibrosis, and upregulate its downstream signaling in fibroblasts [7]. This promotes gene expression of collagen (types I and III) and fibronectin, and suppresses matrix-degrading enzymes, skewing the balance toward matrix accumulation. ROS also modulate fibroblast signaling kinases and transcription factors (such as p38 MAPK, NF-κB, and Smad pathways) that drive the fibrotic program. Studies have shown that oxidative stress not only enhances collagen synthesis but can also alter matrix metalloproteinase activity in cardiac fibroblasts, contributing to maladaptive remodeling [5,7,8]. The net effect is that sustained oxidative stress after injuries like myocardial infarction or in pressure overload conditions leads to interstitial and perivascular fibrosis. This fibrosis increases myocardial stiffness, impairs relaxation (diastolic dysfunction), and can disrupt the synchronized contraction of cardiac muscle. In turn, fibrotic tissue can exacerbate cardiac dysfunction and promote arrhythmias by creating conduction heterogeneity. Notably, ROS has a dual role in remodeling: moderate ROS may initially aid in matrix turnover and wound healing, but high ROS levels clearly favor fibrogenesis over matrix degradation [3]. By triggering fibroblast activation and persistent extracellular matrix deposition, oxidative stress is a key pathological driver of cardiac fibrosis and the resultant decline in cardiac function.

### 3.3. Macrophage-Driven Oxidative Inflammation in Atherosclerosis and Myocardial Infarction

Macrophages are central mediators of inflammation in cardiovascular disease, and their production of ROS can have pathological consequences in both chronic and acute settings. In atherosclerosis, oxidative stress and macrophage function are intimately linked. Circulating monocytes enter the arterial intima and differentiate into macrophages in response to endothelial injury and lipid accumulation. These macrophages are exposed to modified lipids and secrete pro-inflammatory cytokines, creating a cycle of inflammation. A significant source of ROS in developing plaques is the macrophage itself: activated macrophages express NADPH oxidase enzymes (such as NOX2) that actively generate superoxide [4]. The ROS released in the plaque oxidize LDL particles retained in the vessel wall, producing oxidized LDL (oxLDL) [9]. OxLDL is highly pro-inflammatory and is avidly taken up by macrophages through scavenger receptors, turning them into lipid-laden foam cells [9]. Foam cells perpetuate inflammation by releasing more cytokines and ROS, thereby recruiting additional immune cells to the plaque. This oxidative inflammation drives the progression of atherosclerotic lesions and can weaken the fibrous cap by degrading collagen, contributing to plaque instability. Antioxidant defenses in the vessel wall, such as glutathione peroxidase and paraoxonase, normally act to mitigate lipid oxidation; when these are overwhelmed or deficient, atherosclerosis is accelerated. For instance, ApoE^−^/^−^ mice lacking glutathione peroxidase show increased plaque formation, highlighting the protective role of antioxidants in atherogenesis [10,11]. In the context of myocardial infarction (MI), macrophage-driven oxidative stress contributes to both injury and repair. During acute MI, especially upon reperfusion, there is an explosive burst of ROS from infiltrating neutrophils and macrophages, leading to what is known as ischemia–reperfusion injury. ROS produced by these inflammatory cells can extend tissue damage by inducing cardiomyocyte death beyond the initial ischemic area. Experimental studies indicate that reducing macrophage recruitment or activation can limit oxidative injury; for example, mice deficient in the monocyte chemoattractant receptor CCR2 have attenuated oxidative stress and smaller infarcts after ischemia–reperfusion [12]. In the subacute phase after MI, macrophages display a spectrum of phenotypes. Pro-inflammatory (M1-like) macrophages dominate early, releasing ROS and proteases that clear necrotic debris but can also harm viable cells. As healing progresses, anti-inflammatory or reparative (M2-like) macrophages, which produce less ROS and more growth factors, facilitate tissue repair and scar formation. An imbalance with excessive oxidative activity from macrophages can impair the resolution of inflammation and lead to adverse remodeling of the myocardium. Oxidative stress in macrophages also intersects with their signaling functions; for instance, high ROS can activate the NLRP3 inflammasome, leading to interleukin-1β release and further inflammation, which is detrimental post-MI. Conversely, macrophage antioxidants (upregulated via Nrf2 or enzymes like HO-1) help to curb the oxidative inflammatory response and promote healing. In summary, macrophage-driven oxidative stress is a double-edged sword in cardiovascular disease: it underlies the lipid oxidation and inflammation of atherosclerosis and contributes to acute injury in myocardial infarction. It also signals the recruitment and activation of pathways needed for eventual healing. Controlling excessive ROS from macrophages, without abolishing their essential defensive functions, is thus an important therapeutic consideration in treating heart disease.

## 4. Pharmacological Strategies: Antioxidants as Therapeutic Agents

### 4.1. Natural Antioxidants (Polyphenols, Vitamins, Flavonoids) and Their Cardiovascular Benefits

Dietary and natural antioxidants have drawn intense interest for their cardioprotective potential. Epidemiological studies have correlated high intake of antioxidant-rich foods (fruits, vegetables, teas, and wines) with reduced cardiovascular risk, prompting investigations into specific compounds. Polyphenols, a broad class of phytochemicals (including flavonoids, phenolic acids, and stilbenes), are among the most studied natural antioxidants in this context. These compounds can scavenge free radicals and bolster endogenous antioxidant defenses via Nrf2 activation. For example, resveratrol—a polyphenol found in grapes and red wine—has been shown in experimental models to attenuate cardiac oxidative stress and fibrosis [13]. In rodent studies of heart disease, resveratrol supplementation reduced cardiac fibroblast activation and collagen deposition, thereby limiting pathological remodeling. Other polyphenols such as quercetin (from fruits and onions), catechins (from green tea), and curcumin (from turmeric) have demonstrated anti-inflammatory and antioxidant effects that translate into improved cardiac function in animal models of hypertension, ischemia, or cardiotoxicity. Flavonoid-rich extracts from foods have also exhibited benefits: administration of grape seed extract or açai berry, which are rich in anthocyanins and flavonoids, significantly attenuated cardiac remodeling and dysfunction after myocardial infarction in rats [2]. These benefits are attributed to reduced myocardial oxidative damage and inflammation in treated animals. Vitamins with antioxidant properties have likewise been studied. Vitamin C (ascorbic acid) is a water-soluble antioxidant that can neutralize ROS in extracellular fluid and within cells, while vitamin E (α-tocopherol) protects membrane lipids from peroxidation. In experimental cardiomyopathy induced by doxorubicin (a chemotherapy agent causing oxidative cardiac injury), high-dose vitamin C improved left ventricular function and lowered markers of lipid peroxidation [14,15,16]. Vitamin E, often studied in combination with vitamin C, can synergistically maintain redox balance in plasma and membranes. Beta-carotene and other carotenoids can also quench singlet oxygen and may protect against LDL oxidation. Despite strong preclinical evidence and biochemical rationale, the translation of natural antioxidants into clinical cardiovascular benefit has been challenging. Some small clinical trials have reported positive effects—for instance, short-term resveratrol supplementation improved left ventricular function in patients with heart failure or stable coronary disease in pilot studies [2,17,18]. However, large randomized trials of antioxidant vitamin supplements (such as vitamin E or beta-carotene for prevention of heart attacks and strokes) have largely been neutral or disappointing. In one notable trial, a combination of vitamin E and vitamin C did not significantly reduce the incidence of major cardiovascular events or improve outcomes compared to placebo [19]. The complexity of these interventions (optimal dosing, timing, and patient selection), as well as the possibility that antioxidants work best in the context of a whole diet rather than isolated pills, may explain the inconsistent results. Nonetheless, natural antioxidants remain a focus of interest due to their relative safety and the epidemiological links to heart health. Ongoing research is exploring concentrated plant extracts or novel formulations (e.g., nanocarriers of polyphenols) to enhance the bioavailability and efficacy of natural antioxidants in cardiovascular therapy.

### 4.2. Synthetic Antioxidants and Their Clinical Relevance

**Figure 2:**
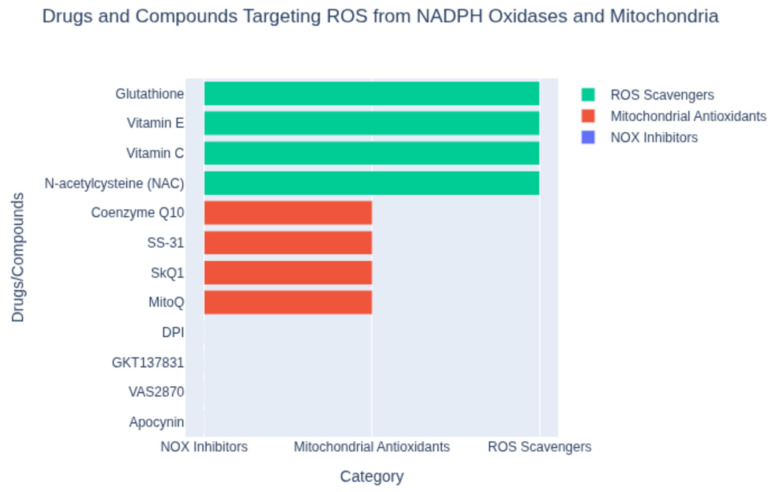
Classification of compounds targeting reactive oxygen species (ROS) from NADPH oxidases and mitochondria. Shown are representative agents grouped by mechanism: NOX inhibitors (e.g., apocynin, VAS2870), mitochondrial antioxidants (e.g., MitoQ, SS-31), and ROS scavengers (e.g., N-acetylcysteine, vitamin C). This classification highlights distinct strategies for mitigating oxidative stress.

Beyond dietary agents, a variety of synthetic or pharmacological antioxidants have been developed to target oxidative stress in cardiovascular disease. These include small-molecule ROS scavengers, enzymes or enzyme mimetics, and drugs repurposed for their antioxidant properties (Figure 2). One classical example is probucol, a synthetic lipid-lowering drug with potent antioxidant activity. Probucol can incorporate into LDL particles and prevent their oxidation, and it also upregulates cholesterol efflux from macrophages. In clinical studies, probucol demonstrated efficacy in reducing atherosclerosis progression and complications. Notably, the Antioxidant Probucol trial showed that probucol significantly reduced the rate of restenosis (re-narrowing of the artery) after coronary angioplasty [19], whereas a multivitamin antioxidant regimen did not yield benefit [19]. This highlighted the potential of targeted antioxidant drugs over generic vitamin supplementation in certain settings. Another synthetic approach has been the use of N-acetylcysteine (NAC) and related thiols to boost intracellular glutathione, a major endogenous antioxidant. NAC has been studied in settings like myocardial infarction and heart failure to combat oxidative stress. A recent trial with a glutathione precursor in patients with acute ST-elevation MI found that it reduced oxidative stress markers (like NADPH oxidase activity) and was associated with improved left ventricular function [20,21], suggesting that enhancing glutathione could be cardioprotective. Enzyme inhibitors that blunt ROS production at the source are also an important category of synthetic antioxidants. Xanthine oxidase inhibitors such as allopurinol and febuxostat, used clinically to treat gout, reduce the formation of superoxide and uric acid from purine metabolism. In animal models of atherosclerosis, allopurinol treatment reduced plaque formation and oxidative stress [22,23]. Some small studies in humans with heart failure or ischemic heart disease have hinted at improved endothelial function or ventricular function with allopurinol, though hard outcome benefits remain unproven. NADPH oxidase inhibitors are another promising strategy, given the pivotal role of NOX enzymes in cardiovascular ROS generation (especially in macrophages and endothelial cells). Experimental NOX inhibitors (e.g., apocynin or more specific NOX2/NOX4 inhibitors) have been shown to reduce oxidative damage and cardiac remodeling in preclinical studies, but specific agents have not yet become routine clinical therapies pending further trials [24]. Mitochondria-targeted antioxidants represent a novel class of synthetic antioxidants designed to accumulate within the powerhouse of the cell. One such compound is MitoQ, which couples a ubiquinone antioxidant to a lipophilic cation to drive its uptake into mitochondria (Figure 2). In preclinical models of heart failure, MitoQ supplementation decreased mitochondrial H_2_O_2_ production and improved mitochondrial respiratory function [25]. While MitoQ did not dramatically improve ejection fraction in all studies, it did reduce hypertrophy and oxidative damage in pressure-overload heart failure models [25]. Human trials of MitoQ have shown improved vascular endothelial function in older adults and are ongoing for heart failure, reflecting cautious optimism for this targeted approach. Other mitochondria-targeted antioxidants (like the peptide SS-31/elamipretide) are also being investigated for ischemia–reperfusion injury and cardiomyopathy [26]. Additionally, novel nanomaterials and drug-delivery systems are emerging to enhance antioxidant therapy. For instance, inflammation-targeted nanoparticles have been created to deliver antioxidants specifically to cardiac macrophages after MI. In a recent experimental study, a mannan-coated nanoparticle loaded with quercetin was taken up by macrophages in the infarcted heart and effectively neutralized intracellular ROS, shifting macrophages toward a reparative phenotype [27]. Treated rats showed reduced inflammation, decreased adverse remodeling, and improved cardiac function post-MI [27]. This kind of cell-targeted antioxidant strategy holds promise to maximize therapeutic effects while minimizing off-target actions.

Finally, there are emerging antidiabetic drugs that exhibit both antioxidant and anti-inflammatory properties as part of their efficacious clinical benefits. For example, the SGLT2 inhibitors (a class of glucose-lowering drugs) have been recommended for treatment of heart failure due to their robust cardioprotective effects via likely mechanisms of lowering cellular NADH/NAD^+^ ratio and activating Nrf2-mediated antioxidant pathways [28,29]. Alongside SGLT2 inhibitors, recent studies have highlighted the beneficial effects of dipeptidyl peptidase 4 inhibitors (DPP-4) and glucagon-like peptide 1 receptor agonists (GLP-1) on overall and cardiovascular mortality of diabetic individuals [30] (Table 1). Similarly, an earlier antidiabetic drug has been shown to decrease markers of oxidative stress (like plasma TBARS) and improve cardiac structure in non-diabetic patients with coronary disease [2,31]. These findings suggest that enhancing antioxidant defenses can be achieved through multiple pharmacological avenues, including metabolic modulation. In summary, a range of synthetic antioxidants—from direct scavengers and enzyme inhibitors to targeted mitochondrial agents and repurposed drugs—have shown potential in ameliorating oxidative damage in the cardiovascular system. Each strategy comes with unique advantages and challenges, and ongoing research is determining how best to integrate these agents into cardiovascular therapy. Table 1 enlists the pharmacological agents as antioxidants with potential mechanisms of action and cardiovascular relevance, while a schematic diagram illustrates oxidative stress and antioxidant mechanisms in cardiovascular health (Figure 1).

### 4.3. Implications for Redox-Disease Modeling Using Cardiac Organoids

Despite strong biological plausibility and encouraging findings from observational studies, randomized clinical trials evaluating antioxidant supplements have largely failed to demonstrate significant cardiovascular benefits. These discrepancies are often attributed to variations in study design, dosage, duration, and participant health behaviors, underscoring the challenges of translating antioxidant efficacy from preclinical models to clinical practice [34]. Notwithstanding, insufficient attention has been paid to the role of redox imbalance—both oxidative and reductive extremes—in disease pathogenesis [35,36]. In our preclinical studies, we demonstrated that transgenic mice overexpressing cardiac-specific missense mutation R120G of the human αB-crystallin gene recapitulate human cardiomyopathy and exhibit reductive stress [37]. This condition is driven by dysregulated glucose-6-phosphate dehydrogenase (G6PD) activity, which enhances the recycling of oxidized glutathione (GSSG) to its reduced form (GSH), alongside elevated expression and activity of G6PD, glutathione reductase, and glutathione peroxidase.

The field is now advancing toward human-relevant platforms, such as disease-specific organoids and 3D tissue models, which offer superior fidelity to human biology. Cardiac organoids, three-dimensional (3D) in vitro constructs derived from human-induced pluripotent stem cells (hiPSCs) [38], offer significant potential for modeling cardiovascular diseases by recapitulating the human heart’s multicellular architecture and redox biology. These systems enable modeling of complex tissue architecture, patient-specific disease phenotypes, and cellular responses to therapeutics—capabilities that traditional animal models lack [39]. Organoid platforms have already proven effective in studying age-related diseases and are increasingly recognized for their utility in drug screening, toxicity testing, and precision medicine [40].

These models effectively simulate oxidative stress, a hallmark of conditions like myocardial infarction, cardiac fibrosis, and atherosclerosis, where reactive oxygen species (ROS) disrupt cellular homeostasis in cardiomyocytes, fibroblasts, and macrophages. For instance, studies demonstrate that cardiac organoids can model ischemic injury by incorporating oxygen-diffusion gradients and norepinephrine stimulation, revealing fibrotic gene expression and extracellular matrix remodeling akin to human ischemic cardiomyopathy [41]. This enables precise investigation of redox-sensitive pathways, such as Nrf2 signaling, which regulates antioxidant enzymes like superoxide dismutase (SOD) and glutathione peroxidase (GPx). Organoids also facilitate testing of antioxidants, such as vitamin E or Panax notoginseng saponins, to mitigate ROS-induced damage, offering insights into therapeutic efficacy [42]. However, limitations in organoid maturation and vascularization hinder their ability to fully replicate adult heart redox dynamics, necessitating advancements in bioengineering to enhance their translational relevance [43]. Recent studies by Guarnieri et al. [44,45] elucidate the critical role of mitochondrial dysfunction in SARS-CoV-2 pathology, offering valuable insights for cardiovascular research. Both studies demonstrate that SARS-CoV-2 infection disrupts mitochondrial oxidative phosphorylation (OXPHOS), leading to elevated mitochondrial reactive oxygen species (mROS), stabilization of hypoxia-inducible factor-1α (HIF-1α), and a metabolic shift toward glycolysis and the pentose phosphate pathway (PPP) to support viral biogenesis. Olali and coworkers further highlight that SARS-CoV-2 proteins, such as M, Orf3a, and Orf9b, interact with host mitochondrial proteins, impairing membrane potential (ΔΨm), electron transport chain activity, and protein import, while inducing apoptosis. These disruptions, coupled with mROS-driven release of mitochondrial DNA (mtDNA), activate innate immunity and inflammation, processes implicated in cardiac pathologies like viral myocarditis, ischemia–reperfusion injury, and heart failure.

Guarnieri and coworkers provide evidence for the therapeutic potential of mitochondrial-targeted antioxidants, using transgenic mitochondrial catalase (mCAT) and the pharmacological antioxidant EUKB in SARS-CoV-2-infected mice. These interventions reduced mROS, dampened HIF-1α activation, restored OXPHOS gene expression, and attenuated inflammation, resulting in reduced tissue damage and improved survival. RNAseq analysis revealed EUKB’s ability to reverse viral-induced metabolic reprogramming, underscoring the role of antioxidants in stabilizing mitochondrial function. Similarly, Guarnieri et al. suggest that targeting metabolic pathways, such as glycolysis and PPP, inhibits viral propagation, implying that mROS-scavenging antioxidants could mitigate pathology. These findings are highly relevant to cardiovascular health, where oxidative stress and mitochondrial dysfunction drive cardiomyocyte injury and maladaptive remodeling.

Cardiac organoids, derived from human pluripotent stem cells, offer a powerful platform to model these SARS-CoV-2-induced mitochondrial and inflammatory changes in a cardiac-specific context. By introducing viral proteins (e.g., Orf9b, Orf3a), hypoxia, or mROS-inducing conditions, organoids can recapitulate the metabolic reprogramming, oxidative stress, and inflammation observed in both studies. Such models enable high-throughput screening of mitochondrial-targeted antioxidants, such as MitoQ or SS-31, to assess their ability to restore OXPHOS, reduce mROS, and mitigate inflammation-driven cardiac damage (Figure 2). Methodologies from these studies, including RNAseq, proteomics, metabolomics, immunohistochemistry, and TUNEL staining, can be adapted to evaluate antioxidant efficacy in organoids, providing quantitative metrics for mitochondrial function, protein interactions, and tissue viability. For instance, assessing HIF-1α expression, mtDNA release, or glycolytic enzyme activity in organoids could elucidate antioxidants’ cardioprotective mechanisms.

These studies collectively underscore the potential of mitochondrial antioxidants to counteract oxidative stress and metabolic dysfunction in cardiovascular diseases. Cardiac organoids, by bridging the gap between in vitro and in vivo systems, offer a human-relevant platform to translate these findings into novel therapies, accelerating the development of antioxidant-based interventions for oxidative stress-driven cardiac pathologies.

### 4.4. Challenges and Future Perspectives in Antioxidant-Based Therapies for Heart Diseases

Despite strong rationales and positive preclinical results, antioxidant therapy in cardiovascular disease has faced significant challenges. A recurring observation is the lack of consistent benefit in large clinical trials targeting oxidative stress [13,18,46]. For example, while animal models of myocardial infarction or heart failure often show improved outcomes with antioxidant supplements, many human trials of vitamins or antioxidant cocktails have failed to demonstrate clear reductions in heart disease events or mortality. This discrepancy highlights several issues. First, the biology of ROS in vivo is complex: simply scavenging ROS indiscriminately may not restore normal signaling and can even blunt some adaptive stress responses. Low levels of ROS are vital for intracellular signaling and host defense, so broad-spectrum antioxidants might disrupt these physiological functions if not carefully dosed. Second, the bioavailability and distribution of antioxidants present challenges. Orally administered antioxidants (like polyphenols or vitamins) may not accumulate in sufficient concentrations in target tissues (such as the heart or atherosclerotic plaque) to exert meaningful effects. The body’s compensatory mechanisms might also reduce the efficacy of chronic antioxidant supplementation; for instance, cells could downregulate their own antioxidant enzyme production in response to exogenous antioxidants. Moreover, many antioxidants have short half-lives or poor stability, making sustained delivery difficult. Another challenge is patient selection and timing. Antioxidant therapy might be most effective in individuals with demonstrable oxidative stress imbalance (e.g., low baseline antioxidant levels or high oxidative markers), or when given at specific stages of disease (such as during acute reperfusion injury). Trials that did not tailor to these factors could dilute potential benefits by including patients who might not need extra antioxidants or by missing the therapeutic window. Indeed, a treatment like an acute intravenous antioxidant at the time of reperfusion in an MI could have a different impact than chronic oral supplementation in stable patients. Additionally, oxidative stress in cardiovascular diseases is often a downstream effect of other abnormalities (hypertension, diabetes, hyperlipidemia, etc.). Thus, solely targeting ROS without addressing root causes may yield limited results. This understanding is guiding new approaches where antioxidant therapy is combined with or incorporated into broader treatment strategies. For instance, modern heart failure management with drugs like ACE inhibitors, beta-blockers, or SGLT2 inhibitors may already reduce oxidative stress indirectly by improving hemodynamics or metabolism [29,47,48]. The incremental benefit of adding a direct antioxidant on top of optimized therapy may be small, which requires very large trials to detect.

## 5. Clinical Biomarkers of Oxidative Stress Using Emerging Technologies

**Figure 3:**
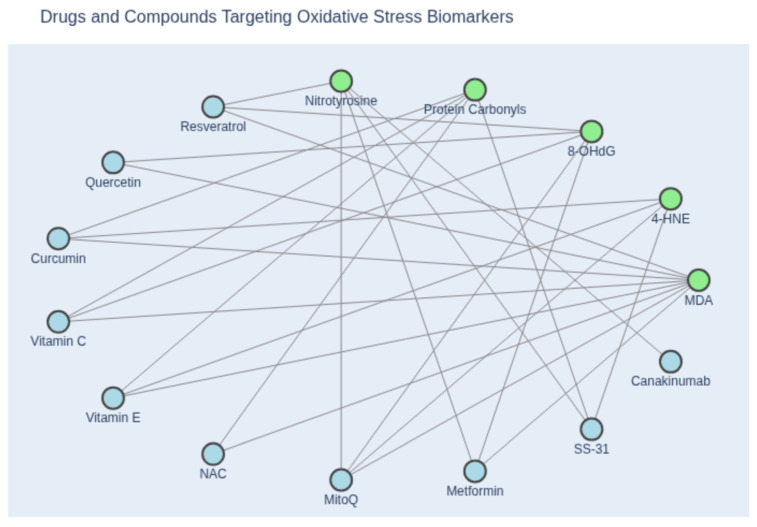
Network diagram illustrating the relationship between oxidative stress biomarkers and inhibitory compounds. Green nodes represent canonical biomarkers of oxidative stress, including malondialdehyde (MDA), 4-hydroxynonenal (4-HNE), 8-hydroxy-2′-deoxyguanosine (8-OHdG), protein carbonyls, and nitrotyrosine. Blue nodes denote pharmacological agents and antioxidants reported to attenuate these markers, based on experimental or clinical evidence. Edges indicate documented inhibitory associations between compounds and specific biomarkers. This visualization highlights the mechanistic diversity of antioxidant interventions and their relevance to redox biology. (Created with Copilot).

Looking forward, the emerging trends in antioxidant therapy will overcome current challenges and offer new avenues for diagnostics [34]. In cardiomyocytes, excessive ROS disrupts contractile function and cell viability, contributing to heart failure and myocardial infarction. Compounds such as 8-hydroxy-2′-deoxyguanosine (8-OHdG) and malondialdehyde (MDA) are commonly elevated, indicating oxidative DNA and lipid damage (Figure 3). In fibroblasts, oxidative stress promotes fibrosis through excessive collagen deposition, often accompanied by increased levels of transforming growth factor-beta (TGF-β) and procollagen peptides, which serve as indirect markers of redox-induced remodeling. Macrophages amplify inflammatory responses, such as atherosclerosis, via ROS-mediated lipid oxidation. Circulating levels of oxidized LDL (oxLDL) and myeloperoxidase (MPO) are key biomarkers reflecting macrophage-driven oxidative activity and vascular inflammation. These cell types not only mediate oxidative damage but also serve as biological targets for antioxidant therapies. Therefore, integrating clinical biomarkers into both experimental and translational frameworks is essential for advancing redox-based diagnostics and therapies. Innovative platforms like 3D organoids may bridge the gap between bench and bedside, offering new avenues for personalized treatment and monitoring of cardiovascular disease. These complementary platforms also offer opportunities to monitor oxidative stress in real time using integrated biosensors and biomarker panels.

By directing antioxidants to the specific cellular sites of damage (such as mitochondria, or inflammatory cells in a plaque or infarct), one can achieve high local efficacy without systemic off-target effects. Mitochondria-targeted antioxidants like MitoQ and SS-31 are examples under clinical evaluation (Table 1). Nanoparticle-based delivery (as demonstrated with macrophage-targeted quercetin in experimental MI) represents another cutting-edge strategy [27]. Such precision medicine approaches could revolutionize how antioxidants are used, turning them from broad dietary supplements into “drugs” that intervene at critical points in the disease process. Another trend is activating endogenous antioxidant pathways rather than supplying antioxidants directly. Compounds that activate Nrf2 (sometimes called “Nrf2 activators” or “indirect antioxidants”) can trigger the cell’s own antioxidant defenses [32]. An example is dimethyl fumarate, an Nrf2 activator already used in multiple sclerosis, which is being explored for cardiovascular benefits due to its ability to induce HO-1 and glutathione-related enzymes. Similarly, natural compounds like sulforaphane (from broccoli) activate Nrf2 and might protect the heart by preconditioning cells against oxidative stress. The timing of such interventions is also a subject of research—a short-term pre-treatment activating Nrf2 could protect against an impending ischemic event (as a pharmacological preconditioning), whereas long-term activation might have anti-inflammatory effects. Combining antioxidant therapy with anti-inflammatory therapy is another future direction, given how closely linked ROS and inflammation are in cardiovascular disease. The success of therapies like IL-1β blockers (e.g., canakinumab) in reducing recurrent heart attacks suggests that dampening the inflammatory-oxidative axis can yield clinical benefits [33]. Antioxidants that specifically target inflammatory cells (like the nanomedicine targeting macrophages) could complement these approaches. Biomarker-guided therapy might also improve outcomes—measuring oxidative stress markers (such as lipid peroxides, oxidized LDL, or glutathione levels) in patients could identify those who are most likely to benefit from antioxidant supplementation, allowing a personalized medicine approach [34].

## 6. Conclusions

Antioxidants play an indispensable role in preserving cardiovascular health by maintaining the delicate redox balance within cardiac cells. In physiological conditions, antioxidant systems in cardiomyocytes, fibroblasts, and macrophages guard against excessive ROS, allowing these species to fulfill normal signaling functions without inflicting damage. In pathological states, however, oxidative stress arises when this balance is upset—contributing to cardiomyocyte injury and death, fibroblast-mediated fibrosis, and macrophage-driven inflammation. These processes underlie many forms of heart disease, from atherosclerotic plaque formation to myocardial infarction and heart failure. Therapeutic interventions with antioxidants have shown that bolstering the heart’s defenses can yield benefits such as reduced infarct size, attenuation of remodeling, and improved endothelial function. Yet, clinical results have been mixed, teaching us that one-size-fits-all antioxidant therapy is unlikely to succeed. The future of antioxidants in cardiovascular medicine lies in targeted, well-timed strategies that complement the body’s own defense mechanisms. Emerging therapies aim to deliver antioxidant effects to the right place at the right time—whether through organelle-specific drugs, immune cell-targeted nanoparticles, or upregulation of endogenous protective pathways. By integrating antioxidant approaches with modern pharmacological treatments and tailoring them to individuals most in need, it may be possible to overcome past limitations. Ongoing research and clinical trials will determine how best to translate the wealth of experimental evidence into tangible health benefits. Importantly, future investigations must incorporate these cutting-edge methodologies and align with recent regulatory shifts, such as the FDA Modernization Act 2.0, which authorizes the use of alternative models—including cell-based assays and computational approaches—for drug safety and efficacy evaluation [49,50].

In closing, antioxidants remain a critical piece of the puzzle in cardiovascular health, and with refined therapeutic approaches, they hold promise in terms of mitigating oxidative injury, slowing disease progression, and improving outcomes for patients with heart disease in the years ahead.

## Figures and Tables

**Table 1:** Summary of Pharmacological agents as antioxidants.

Pharmacological Agent	Mechanism of Action	Cardiovascular Relevance
Resveratrol [2,17,18]	Activates Nrf2 pathway, reduces oxidative stress, inhibits inflammatory signaling	Cardioprotective, reduces cardiac fibrosis and inflammation
Quercetin [27]	Flavonoid antioxidant, scavenges ROS, modulates inflammation, reduces lipid peroxidation	Protects against atherosclerosis, improves endothelial function
Curcumin [32]	Anti-inflammatory, Nrf2 activator, reduces oxidative stress in cardiac cells	Reduces cardiac hypertrophy, protects against ischemia–reperfusion injury
Vitamin C [14,15,16]	Water-soluble antioxidant, neutralizes ROS, enhances endothelial function	Improves vascular health, reduces myocardial oxidative stress
Vitamin E [19]	Lipid-soluble antioxidant, prevents lipid peroxidation, stabilizes cell membranes	Protects against lipid oxidation, supports heart health
Probucol [19]	Inhibits LDL oxidation, upregulates cholesterol efflux, antioxidant activity	Slows atherosclerosis progression, reduces restenosis post-angioplasty
N-Acetylcysteine (NAC)	Boosts glutathione synthesis, reduces oxidative stress and inflammation	Protects against ischemia–reperfusion injury, reduces oxidative damage
Allopurinol [22,23]	Xanthine oxidase inhibitor, reduces uric acid and superoxide production	Reduces vascular oxidative stress, benefits heart failure and ischemic heart disease
Febuxostat [22,23]	More selective xanthine oxidase inhibitor, reduces oxidative stress in vasculature	Reduces oxidative stress in cardiovascular patients, supports endothelial function
MitoQ [25]	Mitochondria-targeted antioxidant, scavenges mitochondrial ROS	Improves mitochondrial health, reduces hypertrophy and heart failure progression
SS-31 [26] (Elamipretide)	Mitochondrial protective peptide, reduces oxidative damage, improves mitochondrial function	Protects cardiac mitochondria, reduces ischemic injury
Apocynin [24]	NADPH oxidase inhibitor, reduces superoxide generation in cardiovascular cells	Attenuates cardiac oxidative stress, reduces inflammation
SGLT2 Inhibitors [28,29]	Reduces oxidative stress via metabolic effects, improves mitochondrial function	Lowers oxidative stress, reduces heart failure risk
Metformin [2,31]	Reduces mitochondrial ROS, activates AMPK, enhances endothelial function	Protects against metabolic and oxidative cardiac stress, improves cardiac function
GLP-1 receptor agonist [30]	Antioxidative properties, reduces endothelial dysfunction	Antidiabetic agent, reduce cardiovascular morbidity and mortality
DPP-4 inhibitors [30]	Antioxidative properties, reduces endothelial dysfunction	Reduction in cardiovascular events, improve CVD health outcomes
Canakinumab [33]	IL-1Î^2^ inhibitor, reduces inflammation and ROS-driven cardiovascular damage	Reduces oxidative inflammation, lowers recurrent cardiovascular events
Dimethyl Fumarate (Nrf2 Activator) [32]	Activates Nrf2 pathway, upregulates endogenous antioxidants, reduces inflammation	Enhances antioxidant response, reduces oxidative stress-driven cardiac remodeling

## Data Availability

This review article contains no new data.
